# Shrimp (*Penaeus vannamei*) survive white spot syndrome virus infection by behavioral fever

**DOI:** 10.1038/s41598-023-45335-5

**Published:** 2023-10-21

**Authors:** Mostafa Rakhshaninejad, Liping Zheng, Hans Nauwynck

**Affiliations:** Laboratory of Virology, Department of Translational Physiology, Infectiology and Public Health, Faculty of Veterinary Medicine, Ghent University, Salisburylaan 133, 9820 Merelbeke, Belgium

**Keywords:** Virus-host interactions, Animal behaviour

## Abstract

Both endotherms and ectotherms may raise their body temperature to limit pathogen infection. Endotherms do this by increasing their basal metabolism; this is called ‘fever’. Ectotherms do this by migrating to warmer places; this is called ‘behavioral fever’. White spot syndrome virus (WSSV) is the most lethal pathogen of cultured shrimp. This study examined the existence of behavioral fever in WSSV-infected *Penaeus vannamei* shrimp. Shrimp weighing 15 ± 0.5 g were inoculated intramuscularly with WSSV and kept in a four-compartment system (4-CS) with all the chambers at 27 °C or with a thermal gradient (27–29–31–33 °C). During the first 4 days post-inoculation, 94% of the WSSV-inoculated shrimp died in the 4-CS with a fixed temperature (27 °C), while only 28% died in the 4-CS with a temperature gradient. The inoculated animals clearly demonstrated a movement towards the warmer compartments, whereas this was not the case with the mock- and non-inoculated animals. With primary lymphoid organ cell cultures, it was demonstrated that the increase of temperature from 27–29 °C to 31–33 °C inhibits virus replication. It is concluded that behavioral fever is used by shrimp to elevate their temperature when infected with WSSV. Behavioral fever prevents WSSV infection and mortality.

## Introduction

Endotherms and ectotherms may raise their body temperature when infected with pathogens to limit their replication. In endotherms, this fundamental reaction to infection is known as fever^1^. It depends mostly on thermogenesis, as well as physiological and behavioral alterations that limit heat loss from the body. Except for a few rare exceptions, ectotherms lack endogenous thermogenesis and retain a body temperature that is very similar to that of their surroundings^2^. When placed in a temperature gradient, non-infected ectotherms choose a specific range of temperature that is preferred for their species^3^. However, if an ectotherm becomes infected or is injected with exogenous pyrogens, they can elevate their body temperature beyond their preferred range by moving towards warmer environments. This phenomenon is referred to as behavioral fever and describes the occurrence of an abrupt increase in final thermal preferendum resulting from an infection^3,4^. Vaughn et al. provided the first description of behavioral fever in ectotherms, showing that desert iguanas (*Dipsosaurus dorsalis*) injected with the inactivated *Aeromonas hydrophila* exhibited a tendency to move towards warmer environments^5^. This behavioral response led to an increase of approximately 2 °C in the body temperature of the iguana^5^. There are examples of behavioral fever in a variety of ectotherms, including vertebrates (reptiles^5–7^, fishes^8–10^, amphibians^11–13^) and invertebrates^14,15^.

The mechanisms behind the behavioral regulation of fever in ectotherms share evolutionary connections with the fever in endotherms^1,3^: (i) exogenous pyrogens act as inducers, (ii) The preoptic area of the hypothalamus has a significant role as a site for integrating pyrogenic signals, and (iii) prostaglandins are effector mediators. Studies have determined that this evolutionary link also encompasses endogenous pyrogens, such as the cytokines that signal the brain when exogenous pyrogens are recognized by immune cells. Cytokines, in particular interleukin 1β (IL-1β), IL6, and interferons in endotherms, and tumor necrosis factor α (TNF-α) in both endotherms and ectotherms have been found to serve as endogenous pyrogens^3,16,17^.

For over a decade, white spot syndrome virus (WSSV) has inflicted disease and mortality on shrimp farms, leading to substantial losses in production. WSSV is a double-stranded (ds) DNA virus that belongs to the family *Nimaviridae* and is characterized as a bacilliform and enveloped virus^18–20^. The mortality rate of shrimp infected with WSSV is 100% within 3–10 days^21^. An infection with WSSV is characterized by lethargy, anorexia, white spots in the carapace, swelling of branchiostegites and reddish discoloration^21,22^.

White spot syndrome (WSS) is the ultimate consequence of host–pathogen–environment interactions^23,24^. Water temperature is regarded to be one of the key environmental elements as it affects shrimp growth, metabolism, survival, oxygen consumption, molting, resistance to toxic compounds and feeding rate^25–27^. Depending on the stage of life and species, shrimp require different temperatures for survival and growth. The optimal temperature for small *P. vannamei* weighing less than 5 g is more than 30 °C, while the optimal temperature for larger shrimp weighing 16 g is about 27 °C^25,28^. The temperature range of 20–30 °C has been found to result in the highest survival of juvenile *P. vannamei*^26^. Juvenile penaeid shrimp exhibit a maximum temperature tolerance of 34–36 °C, beyond which they cannot survive^29^.

The impact of temperature on the results of WSSV infection has previously been recorded. Warmer seasons in tropical regions like Thailand and Ecuador have resulted in a decreased occurrence of WSS^30,31^. In addition, WSSV-infected crustaceans maintained at low (12–15 °C) (crayfish *Astacus astacus*, penaeid prawn *Penaeus japonicus* and/or crayfish *Pacifastacus leniusculus*) or high (> 32 °C) (*Penaeus japonicus* or *Penaeus vannamei*) water temperatures exhibited decreased/deferred death^28,32–34^. It has been shown that temperature has an impact on the results of viral infection in some other ectothermic species, including insects and fish. Examples include infections resulting from a *Bombyx mori* nucleopolyhedrovirus (NPV), a koi herpesvirus (KHV), and a largemouth bass virus (LMBV)^35–37^.

The mechanism by which high water temperature leads to a decline in WSSV-infected *P. vannamei* mortality is not well understood. It has been proposed that elevated temperatures may initiate a host defense reaction that results in the apoptosis of infected cells. Alternatively, it could hamper replication of WSSV^32,38^. A reduction in WSSV DNA content was demonstrated in shrimp infected with WSSV at 32 °C^39^. In *P. leniusculus* hematopoietic stem cells cultured in vitro at temperatures of 4 °C and 16 °C, WSSV replication and the quantity of WSSV DNA significantly decreased^40^.

The objective of this study was to show the occurrence of behavioral fever in shrimp inoculated with WSSV and to evaluate its potential impact on the animal survival. This was accomplished by using a four-compartment system with a thermal gradient to observe the thermal behavior of infected shrimp after virus inoculation. The movement and mortality of the infected shrimp were monitored over a period of time to validate the results. Additionally, the study investigated the effect of elevated temperatures on WSSV replication within an in vitro setting.

## Results

### Effect of incubation temperature on lymphoid organ primary cell viability

**Figure 1:**
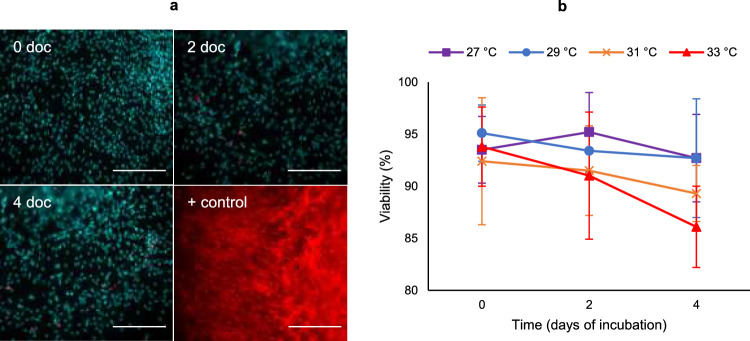
Effect of incubation temperature on lymphoid organ primary cell culture viability. (**a**) Shrimp lymphoid organ primary cells co-stained with Hoechst (blue) and EMA (red) for viability at 0, 2 and 4 days of cultivation (doc) at 33 °C. Positive control: cells killed by incubation at 56 °C for 30 min. Scale bar = 200 μm. (**b**) The percentage of viable shrimp primary lymphoid organ cells at 27 °C, 29 °C, 31 °C and 33 °C at 0, 2 and 4 days of culture. The results are presented as the mean of three independent experiments ± standard deviation (s.d.). One-way analysis of variance (ANOVA) followed by Tukey’s post-hoc test was conducted to determine the statistical significance (n = 36). No significant decrease (F_11,24_ = 1.01, p-value > 0.05) was observed in cell’s viability incubated at non-optimal temperatures (29 °C, 31 °C and 33 °C) compared to the optimal temperature (27 °C) for culturing shrimp primary cells, after 4 days of culture.

The viability of shrimp lymphoid organ primary cells during culturing at different temperatures was evaluated using EMA staining. Shrimp lymphoid organ primary cells were co-stained with EMA and Hoechst for the determination of the cell’s viability at 0, 2 and 4 days post-incubation at 27 °C, 29 °C, 31 °C and 33 °C (Fig. 1). After 4 days of culture, there was no significant decrease (p-value > 0.05) in cell viability incubated at non-optimal temperatures (29 °C, 31 °C and 33 °C) compared to the optimal temperature (27 °C) for culturing shrimp primary cells.

### Effect of incubation temperature on WSSV replication in lymphoid organ primary cells

**Figure 2:**
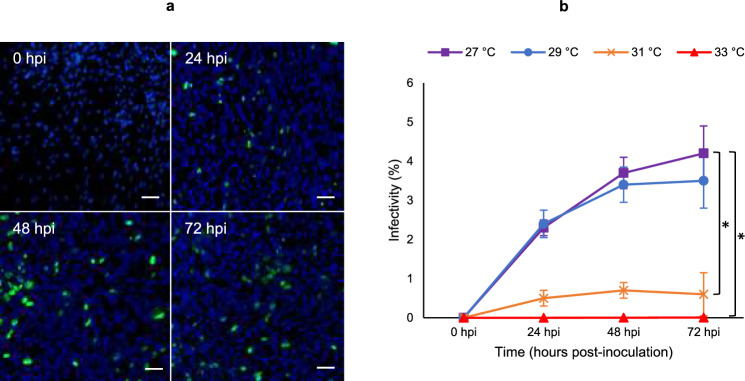
Effect of incubation temperature on WSSV replication in lymphoid organ primary cell culture. (**a**) Indirect immunofluorescence (IIF) staining of WSSV VP28 in primary lymphoid organ cell cultures from *P. vannamei* at 0, 24, 48 and 72 h post inoculation at 27 °C. Blue: Hoechst—staining for nuclei; Green: 1D3 and goat anti-mouse IgG FITC—staining for WSSV VP28. Scale bar = 50 μm. (**b**) The percentage of WSSV-infected shrimp primary lymphoid organ cells incubated at 27 °C, 29 °C, 31 °C and 33 °C at 0, 24, 48 and 72 h post-inoculation. The results are presented as the mean of three independent experiments ± s.d. of the percentage of WSSV-infected shrimp primary lymphoid organ cells incubated at 27 °C, 29 °C, 31 °C and 33 °C over time. One-way analysis of variance (ANOVA) followed by Tukey’s post-hoc test was performed to examine the statistical significance. Asterisks indicate significant differences (*F_15,32_ = 65.5, p-value < 0.05) between 27 °C and the other temperatures at 72 hpi (n = 48; n = 12 at each time point).

The replication of WSSV was evaluated in shrimp lymphoid organ primary cell cultures at different temperatures. The percentage of WSSV infected shrimp primary lymphoid organ cells incubated at 27 °C, 29 °C, 31 °C and 33 °C was assessed at 0, 24, 48 and 72 h post-inoculation (Fig. 2). The percentage of WSSV-infected shrimp primary lymphoid organ cells incubated at 31 °C and 33 °C dropped significantly (p-value < 0.05) compared to the percentage of WSSV-infected cells incubated at 27 °C and 29 °C at 72 h post-inoculation.

### Time course of virus replication in lymphoid organ primary cells as assessed by qPCR

**Figure 3:**
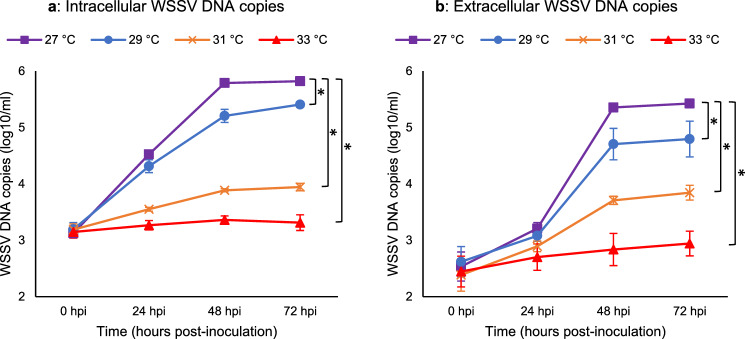
Time course of virus replication in lymphoid organ primary cells as assessed by qPCR. The WSSV DNA copies in the WSSV-infected shrimp primary lymphoid organ cells (intracellular DNA copies (**a**)) and supernatant (extracellular DNA copies (**b**)) incubated at 27 °C, 29 °C, 31 °C and 33 °C at 0, 24, 48 and 72 h post-inoculation. The results are presented as the mean of three independent experiments ± s.d. of the copy number of WSSV genomes in the WSSV-infected shrimp primary lymphoid organ cell cultures incubated at 27 °C, 29 °C, 31 °C and 33 °C over time. Detection limit ≤ 10^1.7^ copies/ml. The statistical significance was determined using one-way analysis of variance (ANOVA) followed by Tukey's post-hoc test. (**a**) Asterisks indicate significant difference (*F_3,44_ = 966.8, p-value < 0.05) between 27 °C and the other temperatures at 72 hpi (n = 192; n = 48 at each time point). (**b**) Asterisks indicate significant difference (*F_3,44_ = 211.2, p-value < 0.05) between 27 °C and the other temperatures at 72 hpi (n = 192; n = 48 at each time point).

The time course of WSSV replication was evaluated by a quantitative PCR in shrimp lymphoid organ primary cell cultures at different temperatures. The copy number of WSSV DNA in infected cells and supernatant of shrimp primary lymphoid organ cultures incubated at 27 °C, 29 °C, 31 °C and 33 °C was assessed at 0, 24, 48 and 72 h post-inoculation (Fig. 3). The copy number of WSSV in both cells and supernatant decreased significantly (p-value < 0.05) at 29 °C, 31 °C and 33 °C compared to the copy number of WSSV at 27 °C at 72 h post-inoculation. No WSSV was found in non-inoculated and mock-inoculated cells.

### Thermal behavior monitoring of juvenile shrimp in response to WSSV infection

**Figure 4:**
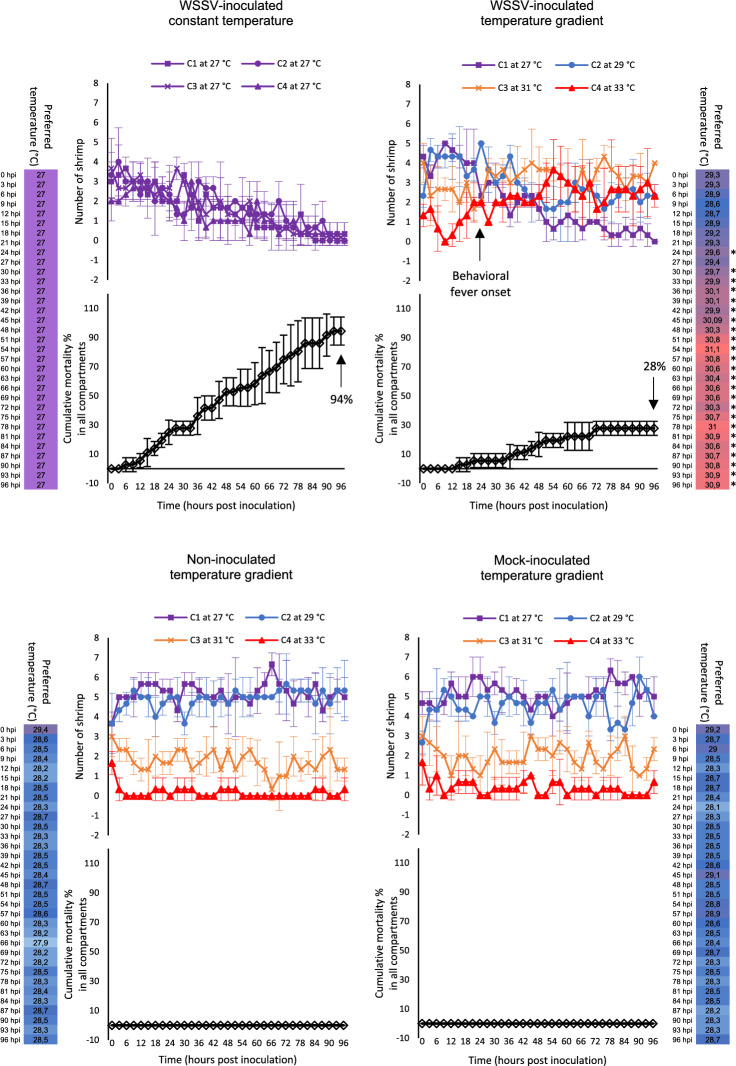
Thermal behavior monitoring in response to WSSV infection in multi-chamber tanks. The distribution, cumulative mortality and preferred temperature of WSSV-inoculated shrimp in the 4-CS kept at 27 °C and of healthy (non-inoculated), mock-inoculated and WSSV-inoculated shrimp in the four compartments reared in the 4-CS with thermal gradient (27–29–31–33 °C). The results for shrimp distribution and mortality are presented as the mean of three independent trials ± s.d. of the number of living and dead shrimp observed every 3 h in each compartment. No mortality was observed among the non-inoculated and mock-inoculated shrimp reared in the 4-CS with a temperature gradient during the first 4 days post-inoculation. 94% mortality was observed among the WSSV-inoculated shrimp reared in the 4-CS with fixed temperature (27 °C) during the first 4 days post-inoculation. 28% mortality was observed among the WSSV-inoculated shrimp reared in the 4-CS with a temperature gradient during the first 4 days post-inoculation. The results for preferred temperature are presented as the means of preferred temperature of the living shrimp at each time point. To determine the statistical significance, a one-way analysis of variance (ANOVA) followed by Tukey's post-hoc test was performed. Asterisks indicate a significant difference (*F_65,231_ = 21.9, p-value < 0.05) in the mean preferred temperature of WSSV-inoculated shrimp kept in the 4-CS with a thermal gradient compared to the mean preferred temperature of non- and mock-inoculated shrimp kept in the 4-CS with a thermal gradient over a period of 96 h post-inoculation (n = 297).

The distribution and mortality of shrimp into the four compartments, either at 27 °C in the four chambers or at a thermal gradient (27–29–31–33 °C) was monitored over time (Fig. 4). A total of 48 healthy shrimp (*Penaeus vannamei*) with an average weight of 15 ± 0.5 g were equally divided into (i) a WSSV-inoculated group kept at 27 °C in the four-compartment system (4-CS) (n = 12) and (ii) a WSSV-inoculated group (n = 12), (iii) a mock-infected group (n = 12), and (iv) a non-inoculated group (n = 12) kept at the thermal gradient. Each trial was repeated three times in order to check the reproducibility of the results. During the first 4 days post-inoculation, the WSSV-inoculated animals spread equally over the compartments when all the compartments were kept at 27 °C. In the case of WSSV-infected animals kept at a temperature gradient, a movement towards the warmer compartments was observed, whereas this was not the case with the mock-inoculated and non-inoculated animals.

WSSV-infected shrimp kept in 4-CS with a thermal gradient showed a higher mean preferred temperature from 28.6 to 30.9 °C when compared to the non- and mock-inoculated shrimp kept in the 4-CS with a thermal gradient at 4 days post-inoculation. Twenty four hours-post inoculation with white spot syndrome virus was recognized as the onset point of behavioral fever in shrimp. Comparing the means of preferred temperatures of WSSV-infected shrimp kept in the 4-CS with a thermal gradient with the means of preferred temperatures of non- and mock-inoculated shrimp kept in the 4-CS with a thermal gradient over time, demonstrated a continuous significant increase in the preferred temperature of shrimp starting at 24 h-post inoculation.

The number of dead shrimp in the different groups (WSSV-infected, mock-infected and non-infected shrimp) and different compartments was monitored by direct observation. During the first 4 days post-inoculation, 94% mortality was observed among the WSSV-infected shrimp reared in the 4-CS with a fixed temperature (27 °C), while only 28% mortality was observed among shrimp reared in the 4-CS with a temperature gradient where they could select the temperature of preference. WSSV-infection in dead animals was confirmed using IIF staining of cephalothorax tissue against WSSV viral protein VP28. No mortality was observed among the mock-inoculated and non-inoculated shrimp reared in the 4-CS with a temperature gradient during the first 4 days post-inoculation.

## Discussion

This study reveals that *Penaeus vannamei* shrimp benefits from behavioral fever in response to white spot syndrome virus infection. Our findings show that behavioral fever increases the survival rate of shrimp by transient movement of shrimp to warmer places. Moreover, the current research shows a direct inhibitory effect of high temperature on WSSV replication under in vitro conditions. Our data highlight the importance of the pathogen-host-environment interplay in aquaculture.

Depending on the environment, WSSV infection can generate extreme mortality up to 100% within 3–10 days^21^. WSSV can replicate in susceptible hosts, such as shrimp, crayfish, and crabs, at temperatures between 16 and 32 °C^28,33,34,40^. It was previously demonstrated in our lab that increasing water temperature from 27 to 33 °C can shut off virus replication and disease/mortality in WSSV infected shrimp in the acute stage of infection before clinical signs appear^28,41,42^. Here we extend these findings by showing the shrimp’s ability to employ thermal preference by moving towards higher temperatures to reduce white spot syndrome virus lethal effects.

Preferred temperature of many animals including endotherms and ectotherms increases after an injection of pyrogenic substances. Our results are in parallel with other studies that have been conducted on behavioral fever in crustaceans. According to Casterlin and Reynolds^43^, injecting inactivated *Aeromonas hydrophila* bacteria into the abdomen or gill chambers of *Cambarus bartoni* crayfish led to a rise of 1.8 °C above the preferred temperature of 22.1 °C. The addition of paracetamol (acetaminophen) to the water inhibited this preference, as reported by Reynolds et al.^44^. Furthermore, when prostaglandin E1 as a fever-producing factor was injected into the haemocoel of *Homarus americanus*, *Cambarus bartoni*, and *Penaeus duorarum*, the preferred temperature increased by 4.7 °C, 3.4 °C, and 4.5 °C, respectively, over a 24-h period^45,46^. It is in agreement with our study that indicated an increase of 2.4 °C in the mean preferred temperature of WSSV-infected shrimp kept in the 4-CS with a thermal gradient at 4 days post-inoculation.

Febrile temperatures can negatively affect the growth of invasive pathogens^47,48^. Our study clearly demonstrates that high incubation temperature inhibited virus DNA replication and synthesis of the envelope protein VP28 in vitro. Mutations in a putative RNA polymerase^35^ or protein kinase-1^49^ have been shown to limit the expression of late viral components, including envelope proteins, in temperature-sensitive mutant baculoviruses at high temperatures. These results suggest that high temperatures may influence enzyme activity of dsDNA viruses during the early replication phase. In the case of WSSV, it is unknown whether high water temperatures impair enzymes. This should be investigated with biochemical tests. Our in vitro study using shrimp lymphoid organ primary cells showed that high temperatures such as 31 °C and 33 °C do not negatively affect the host cells’ viability for at least 4 days while the DNA replication of WSSV clearly decreased. This study confirmed the detrimental influence of the high temperature on WSSV DNA replication.

Earlier studies showed that high temperature has a detrimental effect on WSSV replication in vivo^28,32^. At 33 °C, clinical signs were absent, and mortalities dropped to a zero level^28,42,50^. Besides the direct effect on inhibiting microbial reproduction, the temperature elevation of the body during behavioral fever can also improve the immune system performance by stimulating innate immunity. Apoptosis is considered as an innate immune response that contributes to the invertebrate antiviral response^51^. Granja et al. reported a higher survival rate resulting from a substantial rise in the percentage of apoptotic cells in *Penaeus vannamei* infected with white spot syndrome virus maintained at 32 °C in contrast to infected shrimp maintained at 26 °C^38,39^. It suggests that high water temperature stimulates apoptosis and as a result may reduce viral replication. This would allow the shrimp to survive and control the disease^32,39^. These studies are in agreement with our findings that show an inhibition of virus replication in vivo upon migration towards warmer water. However, considering our in vitro studies on cell viability and WSSV replication, our results undermined the role of apoptosis in reducing WSSV replication. Our results showed that higher temperatures do not significantly affect cell viability for at least 4 days, while higher temperatures significantly hampered the ability of WSSV to replicate. Our finding demonstrated that other factors, beyond host cell death, are involved in the suppression of WSSV infection at higher temperatures. More studies are required to investigate the exact mechanism of the shrimp innate immune system during behavioral fever.

According to Rahman et al., raising the temperature to 33 °C failed to decrease mortality, or at least was less efficient in shrimp that had already been infected with WSSV for 24 h at 27 °C. This is due to the fact that the virus had already become systemic at 24 h post-infection^22,24,42^, causing irreversible tissue damage. Our results demonstrate an early and effective onset of behavioral fever before 24 hpi by active movements towards the higher temperatures before the virus becomes systemic.

**Figure 5:**
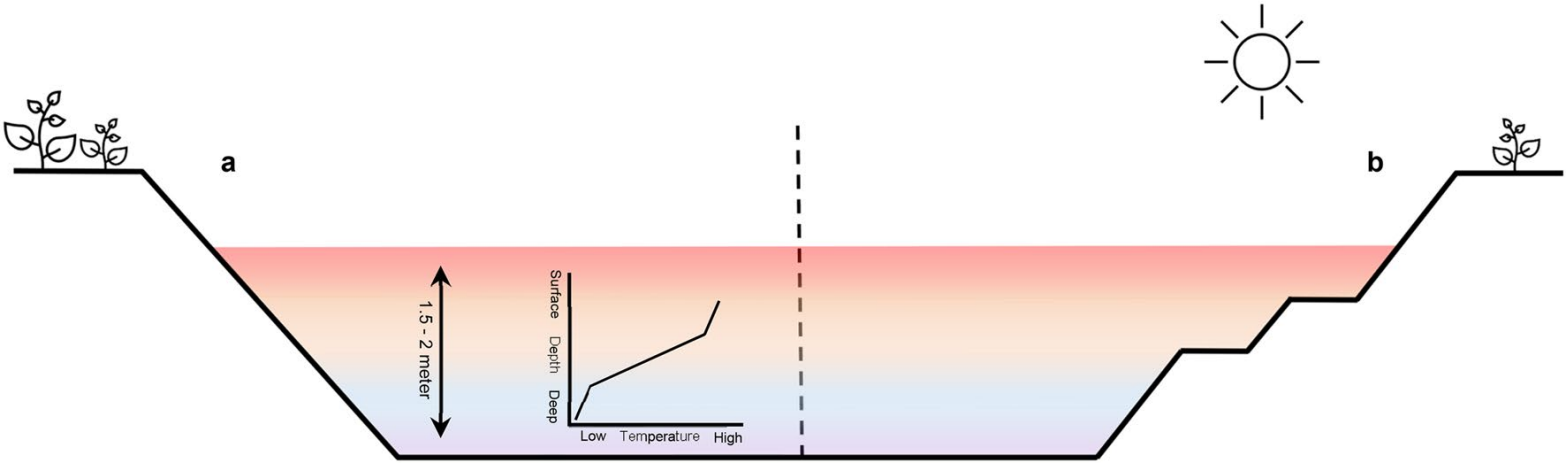
Thermal stratification in a 1.5–2 m deep shrimp pond. (**a**) Conventional border of a shrimp pond. (**b**) New design of a border in a shrimp pond.

Our results show that the preferred temperature in shrimp can be temporarily increased, and that this increase can effectively inhibit viral infection and help the animal to survive. In addition to the benefits of high water temperature in preventing virus infection, high temperature has a negative effect on some environmental variables important for normal shrimp metabolism, such as toxic metabolite concentrations (for example, nitrites or ammonia), dissolved oxygen levels, salinity, and rate of evaporation^52–54^. This study demonstrated that shrimp, by increasing their mean preferred temperature from 29.3 to 30.9 °C, can increase their survival percentage by 66% over a period of 4 days. Our results are important for the design of a shrimp pond. It would be interesting to have several temperature levels at the shore (Fig. 5). This will allow the animal to migrate to the top level with an increased temperature and to stay there to heal from their viral infection. It is important to keep the temperature at the top level between 31 and 33 °C.

Low water temperatures can also reduce the replication of the virus, but this reduction can also lead to an increased susceptibility to other types of infections and diseases^32–34^. Low water temperatures have been shown to reduce the activity of the shrimp's immune system, making them more susceptible to infections and vulnerable to diseases^55,56^. In the future, it would be intriguing to investigate if shrimp can exhibit a different response than behavioral fever (i.e., seeking cooler water) when facing certain pathogens, and if this response can provide an advantage to the infected individual.

In the current study, it is clearly demonstrated that behavioral fever is expressed by shrimp in order to increase the survival capacity against a viral pathogen. However, in invertebrate ectotherms unlike the vertebrate ectotherms, the exact mechanism of behavioral fever onset is not clear. Fever of endotherms and behavioral fever of ectothermic vertebrates share evolutionary conserved mechanisms^3^. The preoptic area (POA) of the vertebrate brain contributes to thermoregulation and fever induction in endothermic vertebrates and behavioral fever of ectothermic vertebrates^57^. In invertebrates, unlike vertebrates, a hypothalamus does not exist. Penaeid shrimp possess a single ventral nerve cord that connects ganglions along their body segments^58^. The peripheral nervous system of crustaceans appears to possess the ability of sensing changes in temperature and adjusting to them through thermal acclimation, without the involvement of the central nervous system's participation in controlling behavior^59^. More research is required in order to investigate the exact mechanism controlling the temperature sensation, thermoregulation and behavioral fever in crustaceans.

In summary, the present study aimed to show that shrimp express behavioral fever in the context of a physiological process driven by behavioral changes that result in increased host survival, primarily through inhibition of virus replication. These findings indicate that behavioral fever, as an innate immune response, can inhibit the virulence of pathogens that infect shrimp. High water temperature at a certain area of the pond may be applied in closed shrimp farming systems to control WSSV. Further research is needed to fully understand the relationship between behavioral fever and different pathogens in shrimp in order to develop strategies for controlling the diseases in the shrimp aquaculture industry.

In conclusion, this study demonstrates that the expression of a febrile response called behavioral fever is used by shrimp to produce an elevated temperature when infected with WSSV by transient migration to warmer areas. Behavioral fever protects them and increases their survival capacity.

## Materials and methods

### Shrimp

SPF *Penaeus vannamei* shrimp weighing 15 ± 0.5 g were received from IMAQUA bv, Lochristi, Belgium. Shrimp were reared for 2 weeks in a recirculation system at the Laboratory of Virology, Faculty of Veterinary Medicine, Ghent University, Belgium. The water temperature was maintained at 27 °C. Sea water with a salinity of 3.2% was made using artificial sea salt (Instant Ocean, Aquarium systems, France) and deionized water. Shrimp were fed 2% of average weight with a commercial shrimp diet every day. Water quality was tested every day by measuring ionized ammonia (Aquamerck, Germany).

### Virus

For this study, the WSSV Thai-1 strain was utilized. The virus was originally obtained from infected *Penaeus monodon* in Thailand, and it was subsequently transmitted to *Pacifastacus leniusculus* crayfish. Gill suspension of crayfish infected with WSSV Thai-1, was kindly provided by K. Söderhäll (Uppsala University, Sweden). The virus was thereafter propagated in specific pathogen-free (SPF) *P. vannamei* in the Laboratory of Virology at the Faculty of Veterinary Medicine, Ghent University, Belgium. In summary, WSSV Thai-1 inoculum was prepared by homogenizing 50 g of moribund WSSV-infected shrimp carcasses without stomach and hepatopancreas in 100 ml of crustacean PBS (371.9 ± 25 mM NaCl, 2.7 mM KCl, 2 mM KH_2_PO_4_, 8 mM Na_2_HPO_4_, with pH: 7.2–7.4 and osmolality: 780 mmol/kg) on ice. The homogenate was diluted 1:10 in crustacean PBS and centrifuged at 3000*g* for 20 min at 4 °C. Then, the supernatant was collected and centrifuged at 13,000*g* for 20 min at 4 °C. The supernatant passed through a 0.45 μm membrane (Sarstedt, Germany) and was stored at − 70 °C^60^. The mock inoculum was prepared from healthy shrimp following the same procedure. Using the method described by Escobedo-Bonilla et al.^60^, the infectivity titers of the viral stocks were investigated. When intramuscularly inoculated into SPF *P. vannamei*, the median infectious titer of the stock was found to be 10^5.8^ SID_50_ (shrimp infectious dose with 50% endpoint) per ml.

### Primary cell cultures from the lymphoid organ

In order to disinfect shrimp for lymphoid organ dissection, they were immersed in a 4.0% hypochlorite solution and then 70% ethanol solution made in seawater (salinity of 3.2%). After 2 min immersion in disinfectants (each for 1 min), the shrimp were rinsed five times with cold sterile seawater. The lymphoid organ, which consists of two small, ovoid-shaped organs located between the stomach’s lateral edge and the anterior side of the hepatopancreas, were dissected and rinsed four times with HaNaMoRa medium (patent application number PCT/EP2023/051493, filing date 23/01/2023) before being cut into 0.5 mm^3^ explants. The explants were then placed into wells of 24-well plates (containing a glass coverslip coated with 0.1% gelatin) with 1 ml of HaNaMoRa cell culture medium per well. The cultures were kept in an incubator at 27 °C and observed daily using an inverted microscope. Every 3 days, half of the culture medium was replaced. To detach and subculture the outgrowing cells after 10 days post seeding, 1.5 mg/ml collagenase IV plus 3 mM CaCl_2_ dissolved in crustacean PBS was used. Following the removal of the explants, the cells were collected by centrifugation at 400*g* for 10 min. The cellular pellet was resuspended in 1 ml of fresh culture medium. Cell counting was done using a Bürker–Türk counting chamber. Finally, 1.1 × 10^5^ cells were seeded in each well of 24-well plates containing a glass coverslip coated with 0.1% gelatin. At 24 h post-seeding, the cell cultures were ready for evaluating the cell’s viability and determining their susceptibility to WSSV infection.

### Viability of cells in lymphoid organ primary cell cultures

The viability of the cells in shrimp lymphoid organ primary cell cultures at different temperatures was evaluated using EMA (Ethidium Monoazide) staining with excitation/emission of approximately 504/600 nm. Sub-cultured cells were incubated at 27 °C, 29 °C, 31 °C or 33 °C and the cell viability was evaluated at 0, 2 and 4 days of cultivation (n = 36; n = 12 at each time point). Cell cultures were subjected to the following steps in order to evaluate the percentage of viable cells using EMA staining: (i) incubation with 200 μl EMA solution (20 μg/ml in medium, Invitrogen) on ice and in the dark for 30 min, (ii) exposure to bright light on ice for 10 min, (iii) fixation with 500 μl of 4% paraformaldehyde for 10 min, (iv) incubation with 200 μl of Hoechst solution (10 μg/ml in PBS, Sigma Aldrich) for 10 min, and (v) rinsing once with PBS and once with ultra-pure water. Fluorescence microscopy was utilized to examine samples (Leica Thunder, Germany).

### Quantitation of cultured cells immunopositive for WSSV VP28 antigen

The WSSV infection in shrimp lymphoid organ primary cell cultures was evaluated at different temperatures using indirect immunofluorescence (IIF) staining. Primary cells were sub-cultured after 10 days of incubation at 27 °C. Sub-cultured cells in 24-well plates (containing a glass coverslip coated with 0.1% gelatin) were inoculated with 250 μl of 10^5.8^ SID_50_ ml^−1^ of WSSV Thai-1 or mock inoculum and were incubated at 27 °C for 1 h. After 1 h of incubation, the inoculum was discarded, and wells were rinsed three times with fresh culture medium. One milliliter of HaNaMoRa medium was added to each well of a 24-well plate and cells were further incubated at different temperatures: 27 °C, 29 °C, 31 °C and 33 °C. The percentage of WSSV infected shrimp primary lymphoid organ cells incubated at 27 °C, 29 °C, 31 °C and 33 °C was assessed at 0, 24, 48 and 72 h post-inoculation. The shrimp lymphoid organ primary cell cultures of non-inoculated (n = 48; n = 12 at each time point), mock-inoculated (n = 48; n = 12 at each time point) and WSSV-inoculated (n = 48; n = 12 at each time point) were fixed using 100% methanol at − 20 °C for 20 min. Cells were rinsed three times for 5 min using PBS and incubated with 3.5 μg ml^−1^ of the monoclonal antibody 1D3 (AbClon Inc., South Korea) targeted against viral antigen VP28^61^ of WSSV for 1 h at 37 °C. Then, cells were rinsed with PBS and incubated with fluorescein isothiocyanate (FITC)-labeled goat anti-mouse IgG (F-2761, Molecular Probes, The Netherlands) for 1 h at 37 °C. Cells were finally rinsed with PBS, washed with ultra-pure water and analyzed by fluorescence microscopy.

### WSSV quantitative PCR with primary cell cultures

The time course of WSSV replication was evaluated by quantitative PCR in shrimp lymphoid organ primary cell cultures at different temperatures. Primary cells were sub-cultured after 10 days of incubation at 27 °C. Sub-cultured cells in 24-well plates (containing a glass coverslip coated with 0.1% gelatin) were inoculated with 250 μl of 10^5.8^ SID_50_ ml^−1^ of WSSV Thai-1 or mock inoculum at 27 °C for 1 h. After three washes with cold culture medium, 1 ml fresh culture medium was added to each well and cells were further incubated at 27 °C, 29 °C, 31 °C and 33 °C. The replication time course of WSSV in non-inoculated (n = 192; n = 48 at each time point), mock-inoculated (n = 192; n = 48 at each time point) and WSSV-inoculated (n = 192; n = 48 at each time point) shrimp primary lymphoid organ cells incubated at 27 °C, 29 °C, 31 °C and 33 °C was assessed at 0, 24, 48 and 72 h post-inoculation. The shrimp lymphoid organ primary cell cultures and supernatant culture medium were used for DNA isolation. VP19 gene was used as target gene to quantitate the number of viral genomes by qPCR at different temperatures and different time points after inoculation.

After centrifugation at 2000*g* for 10 min, the supernatant was retrieved and stored at − 70 °C. Cells were scraped and mixed with those from the centrifuged supernatant at − 70 °C. A QIAamp DNA Mini kit (Qiagen) was used to extract DNA from the cell fraction and supernatant. The Primer3Plus website was used to design primers within a conserved region of the VP19 coding sequence. For each reaction, 20 μl of PCR mixture was applied. Each PCR mixture consisted of 10 μl PrecisionPLUS 2 × qPCR MasterMix with SYBR Green and ROX (PrimerDesign), 6.5 μl DNase/RNase-free H_2_O, 50 nM forward primer 5′-ATTGGTATCCTCGTCCTGGC-3′, 200 nM reverse primer 5′-GTTATCGTTGGCAGTGTCGTC-3′ and 3 μl sample DNA or standard DNA. The enzyme was activated at 95 °C for 2 min, followed by 40 cycles of 15 s at 95 °C and 60 s at 58 °C. In order to obtain first-derivative melting curves, the mixture was heated to 95 °C for 15 s, cooled to 60 °C for 1 min, and then heated back to 95 °C at 0.3 °C increments^62,63^. A StepOnePlus Real-Time PCR system (Applied Biosystems) was used for amplification, monitoring, and melting curve analysis.

In order to prepare DNA standards for generating a standard curve for absolute quantitation, DNA was isolated from the Thai-1 strain of WSSV stock using a QIAamp DNA Mini kit (Qiagen). The VP19 DNA fragment was amplified using the aforementioned primers. A 50 μl PCR reaction including 32.75 μl DNase/RNase-free H_2_O, 10 μl OneTAQ Standard reaction buffer (New England Biolabs), 0.25 μl OneTAQ DNA Polymerase (New England Biolabs), 1 μl of each aforementioned forward and reverse primers, 1 μl dNTPmix and 4 μl DNA was prepared to amplify the VP19 DNA fragment. The PCR protocol was 94 °C for 30 s followed by 35 cycles of 20 s at 94 °C, 20 s at 55 °C and 60 s at 68 °C with a final elongation step for 5 min at 68 °C. Employing agarose gel electrophoresis, fragments with correct length were isolated and cleaned using a Nucleospin Gel and PCR Cleanup kit (Macherey-Nagel). The concentration of DNA was measured utilizing the NanoDrop 2000 system. For generation of the standard curve, tenfold serial dilutions of the DNA were made over a range of 8 log units (10^9^–10^2^) (efficiency: 98.23%; R^2^: 0.99).

### Design of the multi-chamber temperature tank systems

**Figure 6:**
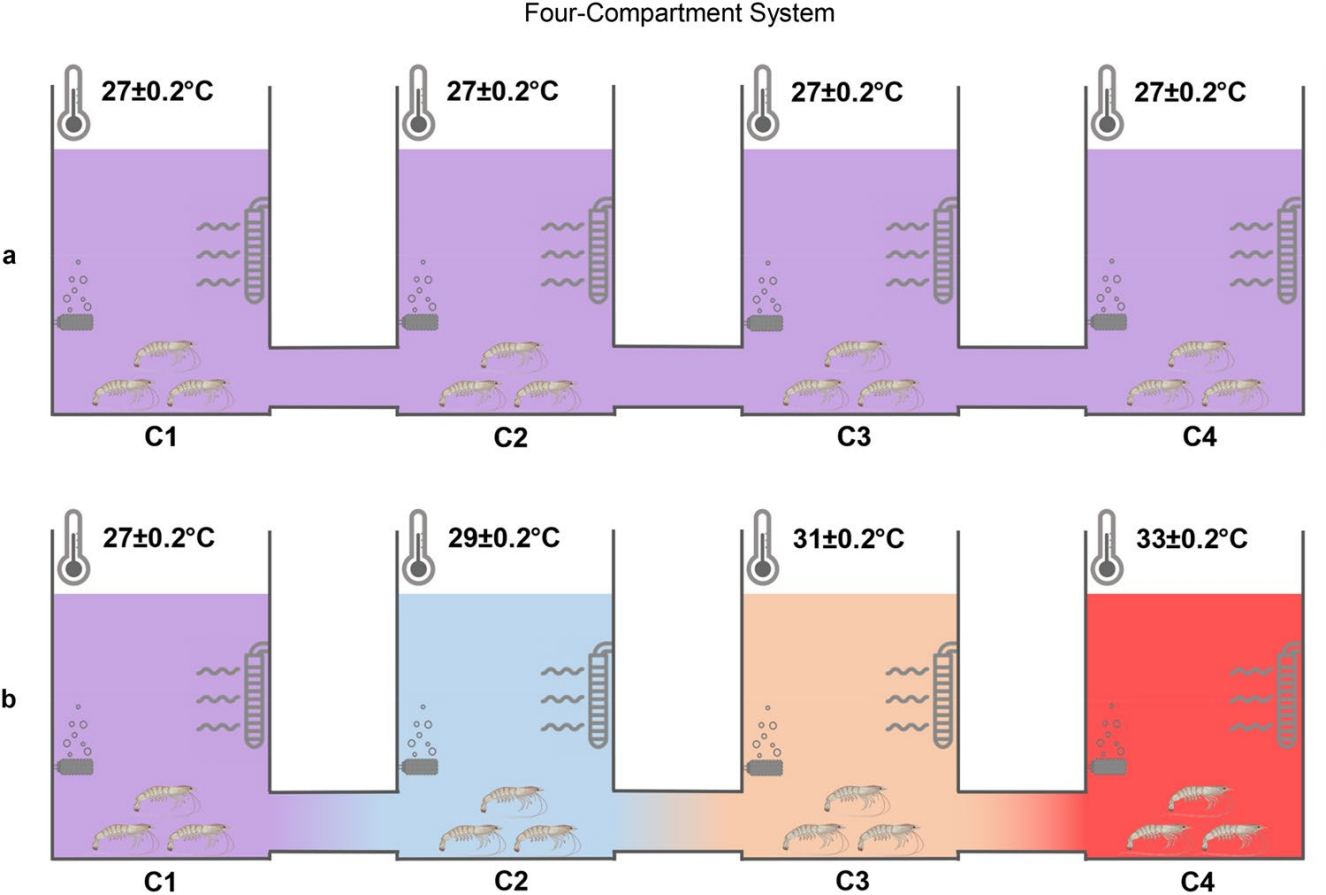
The Four-Compartment System (4-CS) with fixed temperature or with a temperature gradient. 4-CS consisted of four equal tanks of 25 L each (height × diameter: 33 × 31 cm), connected with tubes (length × diameter: 18 × 8 cm) at the bottom of the tanks with all the compartments at 27 °C (**a**) or with a thermal gradient (27 ± 0.2 °C–29 ± 0.2 °C–31 ± 0.2 °C–33 ± 0.2 °C) (**b**).

A four-compartment system (4-CS) was used. The 4-CS consisted of four equal tanks of each 25 L (height × diameter: 33 × 31 cm), connected with tubes (length × diameter: 18 × 8 cm) at the bottom of the tanks (Fig. 6). In the 4-CS, a heater was placed in each tank to keep the desired temperature (EHEIM Thermocontrol, Germany). A thermal gradient (27 ± 0.2–29 ± 0.2–31 ± 0.2–33 ± 0.2 °C) was established between the four chambers by putting a heater in each chamber to ensure the correct temperature in each connected tank. The temperature in all four compartments was controlled by measurements every 3 h. In the 4-CS, daily feeding was performed by equally dividing the feed. Feeding was performed in all compartments at the same time independent of the number of shrimp in each compartment.

### WSSV inoculation in challenge

A total of 48 healthy shrimp (*Penaeus vannamei*) with an average weight of 15 ± 0.5 g were equally divided into a non-infected group, a mock-infected group, a WSSV-infected group kept in temperature gradient and a WSSV-infected group kept at a fixed temperature. In each group, shrimp (n = 12) were initially introduced into the 4-CS with all four chambers at 27 °C. After an acclimatization period of 1 week, the heaters were adjusted in order to produce a temperature gradient (27–29–31–33 °C) or a fixed temperature (27 °C). In the WSSV infection groups, shrimp were intramuscularly injected at the junction between the third and fourth abdominal segments with 50 μl of 10^5.8^ SID_50_ ml^−1^ per shrimp of WSSV Thai-1 strain. In the mock-infected group, 12 shrimp were intramuscularly injected with 50 μl per shrimp of mock inoculum (from healthy shrimp tissue suspension). Each trial was repeated three times in order to check the reproducibility of the results.

### Monitoring of shrimp movements and mortality

Shrimp distribution and mortality of shrimp in the four compartments were monitored over 4 days. Shrimp movements between compartments were monitored every 3 h by direct observation. The results are presented as the mean of three independent trials ± s.d. to determine the number of shrimp occupying each compartment every 3 h.

### Confirmation of WSSV infection (VP28) by histological IIF

In order to confirm WSSV infections in dead animals, an IIF staining against WSSV viral protein VP28 was performed. Dead and moribund shrimp were removed from the compartments every 3 h and covered with ice to slow down the post-mortem changes and to ensure the euthanasia of moribund shrimp. The cephalothoraxes of dead and euthanized shrimp were obtained. The cephalothoraxes were (i) longitudinally dissected, (ii) in 2% methylcellulose embedded and (iii) rapidly frozen at − 70 °C (dry ice and ethanol). 12 μm thick cryosections were generated and fixed in 100% methanol at − 20 °C for 20 min. Following washing sections in PBS (three times for 5 min), sections were incubated with 3.5 μg ml^−1^ of the monoclonal antibody 1D3 (AbClon Inc., South Korea) specifically against the viral antigen VP28 of WSSV^61^ for 1 h at 37 °C. Sections were then rinsed with PBS and treated with fluorescein isothiocyanate (FITC)-labeled goat anti-mouse IgG for 1 h at 37 °C. Finally, sections were rinsed with PBS, washed in ultra-pure water, left to dry, and mounted with a solution containing glycerin and 1, 4-diaza-bicyclo [2,2,2]-octan (DABCO) (ACROS Organics, USA). Sections were examined using fluorescence microscopy.

### Data analysis

IBM SPSS Statistics for Windows, version 26 (Armonk, NY: IBM Corp), was utilized to conduct a one-way analysis of variance (ANOVA) followed by Tukey’s post-hoc test to determine the statistical significance. For all the analyses, a two-sided p-value < 0.05 was considered statistically significant. Normality of the data was verified by the use of the Shapiro–Wilk test. F-ratio and degrees of freedom of each statistical analysis were expressed as F_numerator-df,denominator-df_ = F-value (numerator-df: between-groups degrees of freedom, denominator-df: within-groups degrees of freedom and F-value: mean square between-groups divided by mean square within-groups). Data were given as mean of three independent experiments ± standard deviation (s.d.) at each time point.

## Data Availability

The datasets generated and/or analyzed during the current study are available from the corresponding author on request.
